# Sentinel lymph node biopsy using dye alone method is reliable and accurate even after neo-adjuvant chemotherapy in locally advanced breast cancer - a prospective study

**DOI:** 10.1186/1477-7819-9-19

**Published:** 2011-02-08

**Authors:** Chintamani, Megha Tandon, Ashwani Mishra, Usha Agarwal, Sunita Saxena

**Affiliations:** 1 Department of Surgery, Vardhman Mahavir Medical College, Safdarjang Hospital, New Delhi, 110023, India; 2 Vardhman Mahavir Medical College, Safdarjang Hospital, New Delhi, 110023, India; 3 Indian Council of Medical Research, Institute of Pathology, New Delhi, 110023, India

## Abstract

**Background:**

Sentinel lymph node biopsy (SLNB) is now considered a standard of care in early breast cancers with N0 axillae; however, its role in locally advanced breast cancer (LABC) after neo-adjuvant chemotherapy (NACT) is still being debated. The present study assessed the feasibility, efficacy and accuracy of sentinel lymph node biopsy (SLNB) using "dye alone" (methylene blue) method in patients with LABC following NACT.

**Materials and methods:**

Thirty, biopsy proven cases of LABC that had received three cycles of neo-adjuvant chemotherapy (cyclophosphamide, adriamycin, 5-fluorouracil) were subjected to SLNB (using methylene blue dye) followed by complete axillary lymph node dissection (levels I-III). The sentinel node(s) was/were and the axilla were individually assessed histologically. The SLN accuracy parameters were calculated employing standard definitions. The SLN identification rate in the present study was 100%. The sensitivity of SLNB was 86.6% while the accuracy was 93.3%, which were comparable with other studies done using dual lymphatic mapping method. The SLN was found at level I in all cases and no untoward reaction to methylene blue dye was observed.

**Conclusions:**

This study confirms that SLNB using methylene blue dye as a sole mapping agent is reasonably safe and almost as accurate as dual agent mapping method. It is likely that in the near future, SLNB may become the standard of care and provide a less morbid alternative to routine axillary lymph node dissection even in patients with LABC that have received NACT.

## Introduction

Breast cancer is the most common site specific cancer in women and represents 20% of all female malignancies. In developing countries like India, 25-30% patients still present with locally advanced breast cancers (LABC). The current treatment guidelines for LABC focus upon multimodality approach i.e. neo-adjuvant chemotherapy (NACT) followed by surgery and adjuvant therapies in the form of chemotherapy, radiotherapy, hormone therapy etc. The well known advantages of NACT include, down staging and downsizing of the tumor to make it amenable to breast conservation surgery, as well as serving as an *in-vivo *test of sensitivity to the chemotherapy regimen used [1-3].

The histological status of axillary lymph nodes is one of the most important prognostic factors in patients with breast carcinoma and remains so, even after NACT [1,2]. NACT, initially introduced to downstage LABC to facilitate optimum surgery, results in an improved disease free survival and overall survival, which is comparable with the effects of adjuvant chemotherapy [4-7]. More recently, the indications for NACT have also been extended to selected patients with an early staged disease to allow breast conserving surgery [8,9]. Another potential advantage of NACT is the opportunity to observe chemosenstivity *in vivo*, providing prognostic information [10].

Whether sentinel lymph node biopsy (SLNB) is feasible and accurate following NACT is of significance, since axillary status gets down staged to N0 in considerable proportion of patients after NACT (30-40%). Demonstrating the accuracy of SLNB in this setting can help a proportion of initially node positive patients that have been down staged to N0 by avoiding the morbidity of routine axillary lymph node dissection. The aim of this study was to determine the accuracy of SLNB following NACT for LABC using methylene blue dye alone.

## Methods

The study was conducted in the Department of Surgery, Vardhman Mahavir Medical College Safdarjang Hospital in collaboration with Indian Council of Medical Research, New Delhi, over a period of 1 year (from Dec 2008 to Jan 2009) after obtaining clearance from the "Institutional review Board" and the "Ethical Committee" of Safdarjang Hospital New Delhi India.

### Patients

Thirty fine needle aspiration cytology (FNAC) confirmed cases of LABC (i.e. Stage IIb and stage III) were evaluated after taking informed consent for enrolment in the study. Ultrasonography (USG) and Magnetic resonance imaging (MRI) of both breasts were done for accurate measurement of the basal tumor size in order to stage the disease accurately. A core needle biopsy was routinely performed for baseline tumor marker status and assessing the grade. The FNAC was not used for the nodal metastases in the study. The patients were then subjected to blood and radiological investigations including an echocardiogram before initiation of neo-adjuvant chemotherapy (NACT)that was administered in standard doses at three weekly intervals [Cyclophosphamide 500 mg/m^2^, Adriamycin 50 mg/m^2^(methotrexate in cardiotoxic patients) and 5-FU 500 mg/m^2 ^]. All cases were re-assessed clinically and with ultrasonography and MRI of the breast (using RECIST criteria) for response assessment after each cycle.

After 3 weeks of the last cycle of NACT, the patients were taken up for surgery i.e. modified radical mastectomy (MRM) with a standardized technique by the same surgical team. Intra-operatively peri-tumoral injections of 2-3 ml of methylene blue dye were given followed by breast massage for five minutes before the patient was being draped and prepared for surgery. The sentinel node/s (blue node/s) was mapped and isolated after raising the flaps (the average time taken for the dissection of sentinel node was 10 minutes after injection of the dye). Only nodes that were stained blue were considered as sentinel i.e. even an enlarged or firm axillary node which did not stain was not considered as sentinel. The average number of sentinel nodes removed ranged from one to four. The sentinel lymph node/s was/were sent in a separate container and was/were assessed for the presence of metastatic deposits and compared with the rest of the axillary lymph nodes. As a part of the modified radical mastectomy complete (level-I to level-III) axillary dissection was subsequently performed and the axillary lymph nodes were sent for histopathological evaluation. In all patients, a minimum of ten dissected lymph nodes were considered as optimum axillary dissection.

### Statistical analysis

Thirty patients of locally advanced breast carcinoma were studied using descriptive statistics. The Mc Nemar's Chi square test and paired T test were used to determine association between two variables. P value less than or equal to 0.05 was taken as significant.

The values of the diagnostic parameters related to techniques of SLNB were estimated in terms of sensitivity, specificity, positive predictive value, negative predictive value, false negative rate and accuracy on the basis of distribution of 30 cases into four categories of SLN and axilla expression patterns.

Data analysis was performed by SPSS version 11.5

## Results

All 30 cases were of locally advanced carcinoma (i.e. stage IIb and stage III). The age of the patients ranged from 32-85 years with a mean age of 47.3 years and a standard deviation of 10.98 years(Table 1). Majority of the patients were post-menopausal (20 out of 30 patients i.e. 66.6%).

**Table 1:** Group wise age distribution

Age (years)	No. of patients	Percentage
**31-40**	10	33.3%

**41-50**	12	40%

**51-60**	5	16.6%

**61-70**	3	10%

**71-80**	0	0%

**81-90**	1	3.33%

The distribution of tumor size before and after NACT is shown in Table 2

**Table 2:** Pre NACT vs. Post NACT tumor Size

		Mean	N	Std. Deviation
	**Pre NACT**	6.31	30	2.4
	
**Tumor**	**Post NACT**	3.44	30	1.9

Using the paired t-test for significance, with 95% confidence limits, (p < 0.001) the difference in the pre and post neo-adjuvant chemotherapy tumor size was found to be statistically significant.

56.70% of the patients in our study had clinically N2 disease (fixed ipsilateral axillary nodes), which was in direct correlation with the large tumor size and advanced stage of the disease. However none of the patients had N0 or N3 axilla. After NACT most of the patients were down staged with respect to their axillary lymph node status, with about half (50%) of them having no clinical/sonological/MRI) evidence of lymphadenopathy following NACT(Table 3).

**Table 3:** Lymph Node status before and after NACT Cross tabulation

			Post NACT lymph nodes			Total
				
			N0	N1	N2	
	**N1**	**Count**	9	5	0	14
		
		**% within Pre NACT lymph node**	64.3%	35.7%	0	100%
	
**Pre NACT lymph nodes**	**N2**	**Count**	6	5	5	16
		
		**% within Pre NACT lymph node**	37.4%	31.3%	31.3%	100%

**Total**		**Count**	15	10	5	30
		
		**% within Pre NACT lymph node**	50%	33.3%	16.7%	100%

Out of 14 patients that were N1 before NACT, 9 (64.31%) were down staged to N0, while in 5(35.70%) patients axillary status remained at N1. Out of total 16 patients that were N2 before NACT, 6(37.4%) were down staged to N0. 5(31.3%) were down staged to N1, while in 5 (31.3%) patients there was no change in axillary status.

Using the chi square test (p = 0.049), the difference in pre and post chemotherapy lymph node status was found to be statistically significant.

Relationship of sentinel lymph node biopsy and axillary status is summarized in the Tables 4&5

**Table 4:** Relationship of sentinel lymph node biopsy and axillary status

	Axilla(n = 30)	
	
Sentinel lymph node	Positive	Negative
**Positive**	13(43.3%)	0(0%)

**Negative**	2(6.6%)	15(50%)

**Table 5:** SLNB results by post-NACT axillary status (n = 30)

	POST NACT AXILLARY STATUS					
	
SLNB	N0 = 15		N1 = 10		N2 = 5	
	
	+ve	-ve	+ve	-ve	+ve	-ve
+ve	3	3	4	0	2	1

-ve	0	9	1	5	1	1

N0, N1, N2 is the pre-operative lymph node status, i.e. after NACT, while + represents the histopathological report i.e. pN+/pN-

There were two "false negative cases", one was a "non responder" that was N2 (both pre and post NACT) and the other was a responder (pre NACT-N2 and post NACT-N1) i.e. both false negative cases cases were not N0 after three cycles of NACT. 50% cases i.e. 15/30 cases in our study were down staged from N1 or N2 to N0.

In 13 patients (n = 30) the SLN and the axilla were both positive for the disease while in 2 patients (6.6%), the SLN was negative while the axilla was positive (indicating that the SLNB could not accurately predict the axillary status). In 15 patients (50%, n = 30) both the SLN and the axilla were negative and the SLNB could accurately predict the status of the axilla.

In all patients sentinel lymph node/s was at level I (lateral to Pectoralis minor)

Following parameters were calculated by applying basic descriptive statistical methods:

Sensitivity of SLN = True positives/(true positives +false negatives)

Was found to be = 86.67%

False negative rate = False negatives/(false negatives +true positives)

Was found to be = 13.33%

Negative predictive value = True negative/(true negative + false negative)

Was found to be = 88.23%

Accuracy = True positive + True negative/No. of patients with successfully identified SLN

Was found to be = 93.30%

Sentinel lymph node accuracy parameters were calculated according to standard definitions, used in various studies on sentinel lymph node/s (SLN) and they were as follows:

• The Identification Rate was defined as the number of patients who underwent a successful SLN biopsy divided by total number of patients in whom a SLN biopsy was attempted. The identification ratein the present study was 100% i.e. SLN could be identified in all thirty patients included in the study.

• The results from each successfully identified SLN were categorized as true positives, true negatives, or false negatives, taking the outcome of the complete ALND as **"**reference standard".

• A true negative SLN was defined in this study as a negative SLN and a negative axilla after ALND. The true negatives SLNin the present study were 15(50%).

• A false negative SLN was defined as negative SLN with positive lymph nodes in ALND. There were 2 false negative casesin the present study, out of a total of 15 cases that had a positive axilla after ALND.Of the two "false negative cases", one was a "non responder" that was N2 (both pre and post NACT) and the other was a responder (pre NACT-N2 and post NACT-N1) i.e. Both false negative cases were not N0 after three cycles of NACT. 50% cases i.e. 15/30 cases in our study were down staged from N1 or N2 to N0. 

• A true positive SLN was defined as a positive SLN with or without a positive axilla and in this study 13 caseswere true positives.

Based on these definitions, there were no false positive cases in this study.

Accuracy was computed as the sum of all true positives and true negatives, divided by the total number of patients with a successfully identified SLN. Accuracy in this study was 93.31%.

## Discussion

The histological status of axillary lymph nodes is one of the most important prognostic factors in patients with breast carcinoma and remains so, even after NACT [1,2]. NACT, initially introduced to downstage LABC to facilitate optimum surgery, also results in an improved disease free survival and overall survival, which is comparable with the effects of adjuvant chemotherapy [4-7]. More recently, the indications for NACT have also been extended to selected patients with an early staged disease to allow breast conserving surgery [8,9]. Another potential advantage of NACT is the opportunity to observe chemosenstivity *in vivo*, providing vital prognostic information [10].

Following NACT, traditionally ALND is performed as a part of optimum breast surgery. This however is associated with considerable morbidity [11,12]. A less aggressive approach is therefore sought for, making SLNB after NACT an attractive strategy as the axilla is downstaged to N0 in a number of patients (20-40%) [8,13]. In concordance with the established data, the nodal down staging in the present study was about 50% (in the present study, 9 out of14 N1 and 6 of 16 N2 patients were down staged to N0 i.e. 15/30 patients). Thus considerable number of patients could be spared the morbidity of ALND, once the SLNB gets established as a standard of care in patients with LABC after NACT.

Theoretically, NACT could have several negative effects on the accuracy of the SLN biopsy. Firstly, both primary tumor and metastatic lymph nodes respond by yielding reactive changes like fibrosis affecting the lymphatic drainage patterns. Secondly, chemotherapy can induce an uneven tumor response in axilla. These effects are likely to result in decreased SLNB accuracy after NACT. It has been observed in various studies (Table 6) that there could be a reduction in the identification rates without a significant drop in the predictive value of SLNB even after NACT [14-18]. The accuracy and false negative rates of sentinel lymph node biopsy after NACT were found to be comparable with those of other multicenter trials of SNB (without NACT) and the present study also highlights the same [14-18]. The false negative rates in the present study were 13.3%, favorably comparable with those of (7-13%) in SNB studies before NACT, suggesting that the apprehension regarding skip nodal metastasis could be over-rated and that the SLNB remains almost equally reliable.

**Table 6:** Comparison of identification rates and false negative rates between NSABP-SNB after NACT trial B-27 and three multicenter studies of SNB following breast cancer diagnosis [14-18]

Author	Study Design	No. of patients	Type of lymphatic mapping	Identification rate %	False negative rate%
Mamounas et al [14]	Multicenter SNB after NACT	428	Blue dye Radiocolloid Combination All techniques	78 89 88 85	14 05 09 11

Krag et al [16]	Multicenter SNB before systemic therapy	443	Radiocolloid	93	11

Tafra et al [17]	Multicenter SNB before systemic therapy	529	Combination of blue dye and radiocolloid	87	13

Mc Masters et al [18]	Multicenter SNB before systemic therapy	806	Single agent (blue dye or radio colloid) Combination All techniques	86 90 88	12 6 7

When comparing SLNB success rates amongst heterogeneous studies (i.e. between studies including patients treated with NACT vs. those including patients that have not received NACT), one must take into account the fact that false negative rates depend on the probability of nodal involvement. Among the patients with lower probability of nodal involvement, there is more variation in the false negative rates because the sample size would be smaller [18]. The various single institutional studies evaluating SNB after NACT with their results are summarized in Table 7.

**Table 7:** Single institution series evaluating SNB after NACT [19-25]

Author	Stage	Type of lymphatic mapping	All patients (No.)	Node positive patients	Success rate %	False negative rate%	Conclusion regarding accuracy of SLNB
Breslin TM et al [19,20]	II/III	Blue dye+/_radio colloids	51	25	84.3	12	Accurate

Nason KS et al [21]	T1-T4, N0	Blue dye +radio colloid	15	9	86.7	33	Inaccurate

Julien TB et al [22]	I/II, palpable	Blue dye +/_ radiocolloid	31	6	93.5	0.0	Accurate

Steams V et al [23]	Locally advanced	Vital blue dye	34	13	85.3	14.3	Accurate

Fernandez A [24]	T1-T4. N0-N1	Radio-colloid	40	16	85	25	Inaccurate

Haid A et al [25]	T1-T3, operable	Blue dye+ radiocolloid	33	18	87.9	0.0	Accurate

Miller AR et al [26]	operable	Radicolloid, blue dye, both	35	9	86	0.0	Accurate

All studies combined			230	96	86	12.5	

The largest cohort study till date evaluating SNB after NACT was NSABP B-27 multi-centric randomized trial (N = 428), reported an identification rate, a false negative rate and accuracy of 85%, 11%, and 96% respectively but the locally advanced breast cancers were not included in this study[14]. The overall success rates for sentinel node identification were 84.4% which were similar to results from other single institutional studies [19-26]. This study also concluded that these rates are comparable to those obtained from other multi-centric studies evaluating SLNB and suggested that SLNB is feasible and reliable following NACT. This was also observed in the meta-analysis by Xing and colleagues [15]. In the present study, an identification rate of 100%, false negative rates of 13.30% and accuracy of 93.31% were achieved. The rates do not differ substantially from prior multi-centric studies evaluating sentinel node success rates without NACT, that have reported an identification rate of 88-97% and false negative rates of 5-10%.

As summarized in Table 7, various single institutional studies have examined the efficacy of SLNB after NACT in patients with operable as well as locally advanced breast cancers and reported an identification rates between 84 and 94% [19-21]. All these studies report a higher identification rates when the dual mapping method (i.e. radio-active colloid in combination with blue dye) was used rather than any of the methods used alone. The identification rate in the present study was 100% using the methylene blue dye alone and all the lymph nodes were identified at level-I, highlighting that even after NACT, lymphatic drainage remained more or less predictable [19-25].

The false negative rates in these studies were quiet variable (0-33%), leading to different conclusions about the accuracy of the procedure in this setting. However the small size of these studies can easily account for the wide variability of the estimates. When one examines all these studies after combining the outcomes, the false negative rates would be 12.5%, comparable to our experience and also to the rates seen in studies of SNB before NACT. The false negative rates in our study were 13.30%. There were two "false negative cases", one was a "non responder" that was N2 (both pre and post NACT) and the other was a responder (pre NACT-N2 and post NACT-N1) i.e. both false negative cases were not N0 after three cycles of NACT. Half the cases (50%) in the present study were down staged from N1 or N2 to N0.

There are several limitations as well as strengths in the present study. Limitations include smaller size and higher variability in the age distribution of the cohort. Strengths include the fact that the data was collected prospectively and the same operating team ensured that there was a uniform procedure for lymphatic mapping. There was also a standardized procedure for pathological assessment of the SLN and the axilla by a single team.

## Conclusions

The present study confirms the observations of various other studies in the literature that sentinel lymph node biopsy is feasible and reliable even in locally advanced carcinoma after NACT. The possibility of skip metastasis is perhaps an exaggerated apprehension. There is a high likelihood in near future of SLNB becoming the standard of care even in post NACT-N0 axillae in LABC. SLNB with methylene blue "dye alone" method used in the present study was found to be a cost effective, reliable and almost as accurate as dual agent mapping method to assess the status of axilla. Should SNB become established as the standard method for staging axilla, it will be reasonable to utilize this technique in LABC patients also that have received NACT, expanding the utility of both interventions.

## Competing interests

The authors declare that they have no competing interests.

## Authors' contributions

C, MT, UA, AM and SS contributed to the designing of the study and preparation of manuscript. All authors read and approved the manuscript.
